# Whole Genome Linkage Scan Identifies a Novel Locus on 3q28 for TG/HDL-C Ratio Change Over Time

**DOI:** 10.1093/geroni/igaa057.1591

**Published:** 2020-12-16

**Authors:** Ping An, Allison Kuipers, Iva Miljkovic, Joseph Zmuda, Michael Province

**Affiliations:** 1 Washington University School of Medicine, Chesterfield, Missouri, United States; 2 University of Pittsburgh, Pittsburgh, Pennsylvania, United States; 3 Washington University School of Medicine, SAINT LOUIS, Missouri, United States

## Abstract

TG/HDL-C ratio (THR) represents a single inherited surrogate predictor of hyperinsulinemia or insulin resistance that is associated with premature aging processes, risk of diabetes and increased mortality. To identify genetic loci for THR change over time (ΔTHR), we conducted a whole genome linkage scan among subjects of European ancestry who had complete data from two exams collected about seven years apart from the Long Life Family Study (LLFS, n=3091), a study with familial clustering of exceptional longevity in the US and Denmark. Subjects with diabetes or using medications for dyslipidemia were excluded from this analysis. ΔTHR was derived using growth curve modeling, and adjusted for age, sex, PCs, familial membership, and then log-transformed to approximate normality. Our linkage scan was built on haplotype-based IBD estimation with 0.5 cM average spacing. Heritability of ΔTHR was moderate (46%), and evidence for significant linkage (LOD>3) was identified on 3q28 (LOD=4.1). This locus harbors ADIPOQ among several other promising candidate genes. Interestingly, several studies previously reported suggestive evidence of linkage at this locus for relevant traits including adiponectin, dementia, AD and SBP. This linkage signal was not explained by significant GWAS SNPs for LPL or those under the peak (LOD attenuated to 3.7). In conclusion, we found a novel genetic locus on 3q28 for ΔTHR in subjects without diabetes selected for exceptional survival and healthy aging. Further query of sequence elements including rare functional and regulatory variants at this locus is underway which may reveal novel insights on insulin resistance mechanisms for aging.

